# Supplementary figures and images for: GBP2 as a potential prognostic biomarker in pancreatic adenocarcinoma
Source: PeerJ. 2021 May 11;9:e11423. doi: 10.7717/peerj.11423 (PMC8121056; doi:10.7717/peerj.11423)

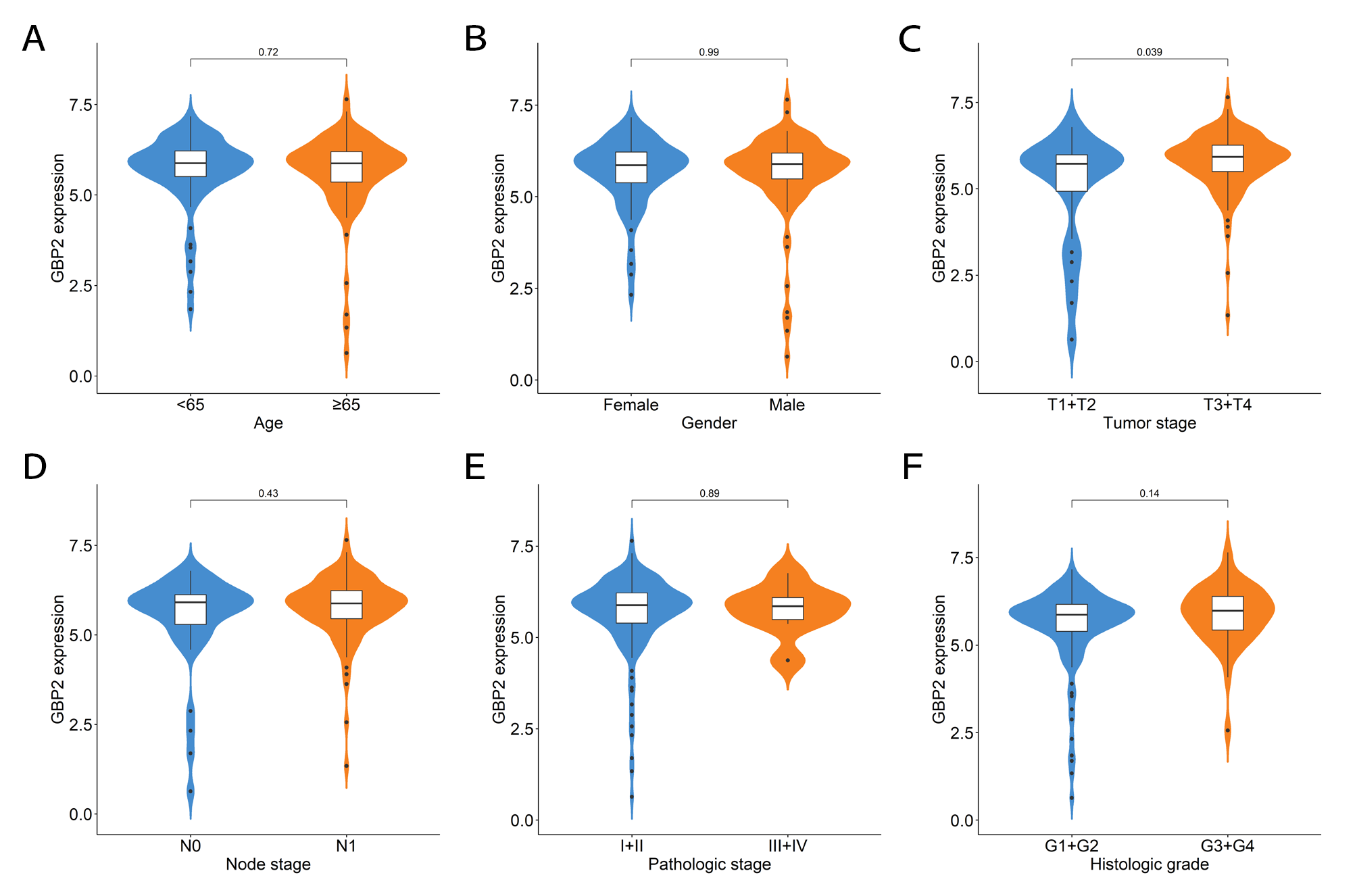

Supplement: Figure S1 [file peerj-09-11423-s001.png]

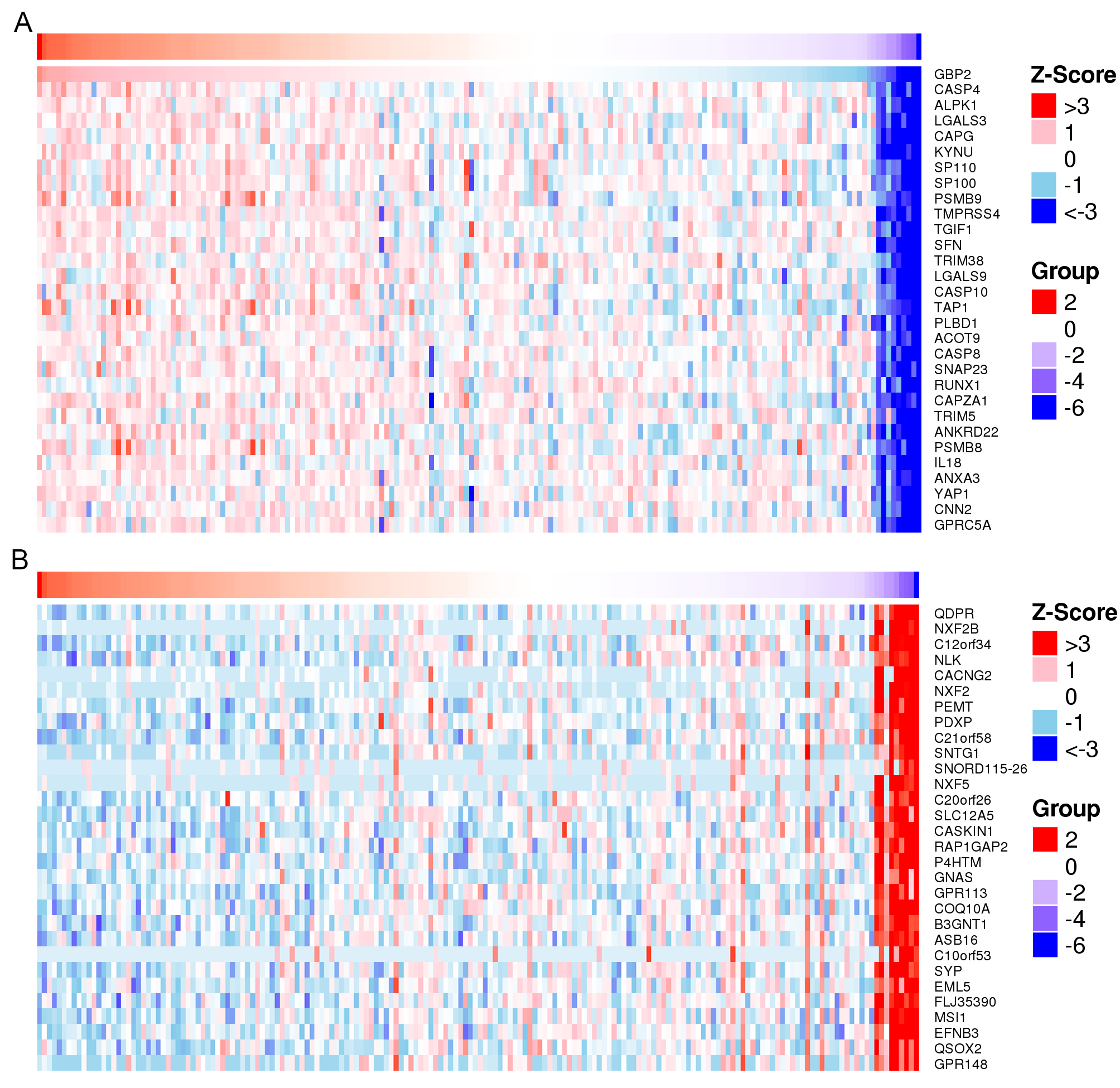

Supplement: Figure S2 [file peerj-09-11423-s002.png]
