# Supplementary material for: GBP2 as a potential prognostic biomarker in pancreatic adenocarcinoma
Source: PeerJ. 2021 May 11;9:e11423. doi: 10.7717/peerj.11423 (PMC8121056; doi:10.7717/peerj.11423)
Supplement: Table S1 [file peerj-09-11423-s003.docx]

| **Dataset** | **Normal** | **Tumor** | **Platform** |
| --- | --- | --- | --- |
| GSE15471 | 39 | 39 | GPL570 |
| GSE60979 | 12 | 49 | GPL14550 |
| GSE62165 | 13 | 118 | GPL13667 |
| GSE91035 | 8 | 25 | GPL22763 |
| GSE102238 | 50 | 50 | GPL19072 |

Supplemental Table S1. Characteristics of the five datasets in this study.
