# Supplementary material for: GBP2 as a potential prognostic biomarker in pancreatic adenocarcinoma
Source: PeerJ. 2021 May 11;9:e11423. doi: 10.7717/peerj.11423 (PMC8121056; doi:10.7717/peerj.11423)
Supplement: Table S2 [file peerj-09-11423-s004.docx]

Supplemental Table S2. The top 20 GO terms from the co-expressed genes of GBP2 enrichment analysis for biological processes (BP).

| **ID** | **Description** | **Adjusted *P* value** | **Gene count** |
| --- | --- | --- | --- |
| GO:0034341 | response to interferon-gamma | 1.46E-10 | 20 |
| GO:0071346 | cellular response to interferon-gamma | 1.48E-08 | 17 |
| GO:0043123 | positive regulation of I-kappaB kinase/NF-kappaB signaling | 1.48E-08 | 17 |
| GO:0043122 | regulation of I-kappaB kinase/NF-kappaB signaling | 6.57E-08 | 18 |
| GO:0007249 | I-kappaB kinase/NF-kappaB signaling | 6.57E-08 | 19 |
| GO:0060333 | interferon-gamma-mediated signaling pathway | 1.35E-07 | 12 |
| GO:2000116 | regulation of cysteine-type endopeptidase activity | 4.20E-07 | 17 |
| GO:0009615 | response to virus | 1.04E-06 | 19 |
| GO:0042119 | neutrophil activation | 1.64E-06 | 23 |
| GO:0002237 | response to molecule of bacterial origin | 1.91E-06 | 19 |
| GO:0043312 | neutrophil degranulation | 4.10E-06 | 22 |
| GO:0002283 | neutrophil activation involved in immune response | 4.20E-06 | 22 |
| GO:0032496 | response to lipopolysaccharide | 4.75E-06 | 18 |
| GO:0002446 | neutrophil mediated immunity | 5.36E-06 | 22 |
| GO:0052548 | regulation of endopeptidase activity | 7.73E-06 | 20 |
| GO:0043901 | negative regulation of multi-organism process | 8.30E-06 | 13 |
| GO:0051607 | defense response to virus | 8.70E-06 | 15 |
| GO:0030217 | T cell differentiation | 9.18E-06 | 15 |
| GO:0043281 | regulation of cysteine-type endopeptidase activity involved in apoptotic process | 1.47E-05 | 14 |
| GO:0045088 | regulation of innate immune response | 1.51E-05 | 20 |
