# Supplementary material for: GBP2 as a potential prognostic biomarker in pancreatic adenocarcinoma
Source: PeerJ. 2021 May 11;9:e11423. doi: 10.7717/peerj.11423 (PMC8121056; doi:10.7717/peerj.11423)
Supplement: Table S3 [file peerj-09-11423-s005.docx]

Supplemental Table S3. The result of KEGG pathway analysis of DEIRGs (ranked by p value).

| **ID** | **Description** | **Adjusted p value** | **Gene count** | **Gene symbol** |
| --- | --- | --- | --- | --- |
| hsa04621 | NOD-like receptor signaling pathway | 9.43E-06 | 15 | GBP2/CASP4/CASP8/IL18/GBP1/CARD6/RIPK2/ANTXR2/BIRC3/OAS2/RIPK3/MYD88/CARD16/IFI16/CASP1 |
| hsa05132 | Salmonella infection | 0.000245 | 14 | CASP4/CASP8/IL18/RIPK2/MLKL/ANXA2/BIRC3/RRAS/RIPK3/MYD88/SNX33/CASP1/TNFSF10/TNFRSF10A |
| hsa05200 | Pathways in cancer | 0.03241 | 18 | CASP8/RUNX1/LAMB3/STAT6/IL4R/GSTP1/TGFA/IL15RA/CDK2/BIRC3/RALB/IL2RG/WNT2/MET/NOTCH2/GLI2/RAC2/MGST1 |
| hsa05130 | Pathogenic Escherichia coli infection | 0.03241 | 10 | CASP4/CASP8/IL18/MYO1E/MYD88/CASP1/TUBA1C/TNFSF10/CLDN1/TNFRSF10A |
