# Supplementary material for: GBP2 as a potential prognostic biomarker in pancreatic adenocarcinoma
Source: PeerJ. 2021 May 11;9:e11423. doi: 10.7717/peerj.11423 (PMC8121056; doi:10.7717/peerj.11423)
Supplement: Table S4 [file peerj-09-11423-s006.docx]

Supplemental Table S4. The top 10 items of GSEA result ranked by P value.

| **ID** | **setSize** | **enrichmentScore** | **NES** | **Adjusted *P* value** |
| --- | --- | --- | --- | --- |
| CHEMOKINE_SIGNALING_PATHWAY | 184 | 0.530627867 | 1.775885 | 0.005585 |
| CYTOKINE_CYTOKINE_RECEPTOR_INTERACTION | 236 | 0.631790193 | 2.135304 | 0.005585 |
| FOCAL_ADHESION | 199 | 0.536102348 | 1.797216 | 0.005585 |
| PATHWAYS_IN_CANCER | 320 | 0.46908673 | 1.60416 | 0.005585 |
| REGULATION_OF_ACTIN_CYTOSKELETON | 206 | 0.481913503 | 1.616779 | 0.005585 |
| AXON_GUIDANCE | 128 | 0.46591804 | 1.531217 | 0.005585 |
| CELL_ADHESION_MOLECULES_CAMS | 129 | 0.613745804 | 2.018818 | 0.005585 |
| JAK_STAT_SIGNALING_PATHWAY | 132 | 0.46784883 | 1.542125 | 0.005585 |
| LEUKOCYTE_TRANSENDOTHELIAL_MIGRATION | 113 | 0.54780324 | 1.790304 | 0.005585 |
| NATURAL_KILLER_CELL_MEDIATED_CYTOTOXICITY | 115 | 0.587425203 | 1.920187 | 0.005585 |
